# The Nutrigenomic Effect of Mela Rosa Marchigiana Callus Extract on Cellular Senescence: Insight From a Preliminary In Vitro Study

**DOI:** 10.1002/mnfr.70336

**Published:** 2025-11-22

**Authors:** Chiara Rucci, Enrica Sordini, Giuseppe Persico, Eugenia Ciurlia, Noemi Pappagallo, Giulia Matacchione, Marco Giorgio, Daniele Fraternale, Maria Cristina Albertini, Dale Annear, Peter De Rijk, Tim De Pooter, Mojca Strazisar, Wim Vanden Berghe, Laura Bordoni, Stefano Amatori, Rosita Gabbianelli

**Affiliations:** ^1^ School of Advanced Studies University of Camerino Camerino, MC Italy; ^2^ Unit of Molecular Biology and Nutrigenomics, School of Pharmacy University of Camerino Camerino, MC Italy; ^3^ Molecular Pathology Laboratory “PaoLa” Department of Biomolecular Sciences University of Urbino Carlo Bo Fano, PU Italy; ^4^ Department of Biomedical Sciences University of Padova Padova Italy; ^5^ Department of Biomolecular Sciences, DISB University of Urbino Carlo Bo Urbino, PU Italy; ^6^ Center of Clinical Pathology and Innovative Therapy, IRCCS INRCA Ancona Italy; ^7^ Center of Medical Genetics University of Antwerp Antwerp Belgium; ^8^ Neuromics Support Facility, VIB Center for Molecular Neurology Antwerp Belgium; ^9^ Department of Biomedical Sciences University of Antwerp Antwerp Belgium; ^10^ Department of Biomedical Sciences, Cell Death Epigenetic Signaling Lab University of Antwerp Antwerp Belgium

**Keywords:** anti‐aging, anti‐inflammatory, DNA methylation, Mela Rosa Marchigiana, transcriptomic analysis

## Abstract

Mela Rosa Marchigiana (MRM) is an apple variety cultured in the center of Italy. Calluses derived from in vitro culture of MRM explants were used to obtain an ethanolic extract rich in pentacyclic triterpenic acids with putative anti‐inflammatory and anti‐aging effects. In this study, we investigated the transcriptomic and epigenetic effects of MRM callus extract (MRME) in an in vitro model of cellular senescence. Senescent HUVECs (sHUVECs) were treated with MRME. Transcriptomic analysis was performed to compare young HUVECs (yHUVECs) with sHUVECs and to evaluate the MRME's effect on sHUVECs. Results show that senescence induces major changes in the transcriptome of HUVECs. MRME downregulates TNF‐α signaling genes in sHUVECs restoring the expression to that observed in yHUVECs. Genome‐wide DNA methylation analysis performed using the Oxford‐Nanopore sequencing platform did not reveal significant changes in DNA methylation levels induced by MRME. Our preliminary results provide additional evidence suggesting that MRME exerts anti‐inflammatory and anti‐aging effects, which may be mediated by the modulation of the expression of inflammaging genes via mechanisms independent of DNA methylation. These findings highlight MRME as a promising candidate for further in vivo studies aimed at exploring its clinical translational potential in counteracting inflammaging.

## Graphical abstract

Mela Rosa Marchigiana (MRM) is an ancient apple variety peculiar of the center of Italy. The in vitro culture of the MRM pulp explants leaded to the production of calluses whose ethanolic extract has been suggested to be rich in pentacyclic triterpenic acids. In this study we investigated the transcriptomic and epigenetic effect of Mela Rosa Marchigiana callus extract (MRME) using in an in vitro model of cellular senescence. Interestingly, our preliminary results suggest that MRME exerts anti‐inflammatory and anti‐aging effects, throughout the modulation of the expression of inflammaging genes via mechanisms independent of DNA methylation.

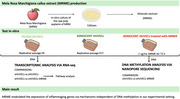

**Abbreviations**
AGE: agarose electrophoresis
cPD: cumulative population doubling
CPM: counts per million
DEGs: differentially expressed genes
DMSO: dimethyl sulfoxide
EMT: E2F targets, G2M checkpoint, epithelial–mesenchymal transition
FC: fold change
FDR: false discovery rate
HUVECs: human umbilical vein endothelial cells
LPS: lipopolysaccharide
MDS: multidimensional scaling
MRM: Mela Rosa Marchigiana
MRME: Mela Rosa Marchigiana callus ethanolic extract
MSigDB: Molecular Signatures Database
MTT: (3‐(4,5‐dimethylthiazol‐2‐yl)‐2,5‐diphenyltetrazolium bromide)
PE: paired‐end
PTAs: pentacyclic triterpenic acids
SA: senescence‐associated
SASP: senescence‐associated secretory phenotype
sHUVECs: senescent human umbilical vein endothelial cells
sHUVECs MRME: senescent HUVECs treated with Mela Rosa Marchigiana callus ethanolic extract

## 1 Introduction

Mela Rosa Marchigiana (MRM) is an ancient apple variety peculiar to the center of Italy [1]. It is characterized by the small size, flat shape, green peel with pink reddish colors, and an intense sweet taste [1]. The plant (*Malus* × *domestica* Borkh, Rosaceae family) is found at altitudes of 400–900 m above the sea level in the Marche region (Italy), where it thrives under the Apennine climatic conditions characteristic of this area.

MRM has been cultivated in the Sibillini region for long, but its production decreased in the past decades until it ceased due to low market demands [2]. However, the productions have recently restarted thanks to the increasing interest in the properties of this variety of fruit. MRM, in fact, has been demonstrated to be an important source of bioactive compounds, and it has been suggested as potentially useful to produce nutraceuticals and food supplements [1, 2, 3]. MRM contains polyphenols such as flavan‐3‐ols/procyanidins, dihydrochalcones, flavonols, and hydroxycinnamic acids, as well as triterpenic acids such as annurcoic acid [1]. Pentacyclic triterpenic acids (PTAs) are a class of secondary metabolites present in several medicinal plants and in a great variety of fruit and vegetables [4]. Recently, PTAs have garnered increasing interest due to their potentiality in promoting human health despite their limited availability in food and plants. The presence of secondary metabolites in fresh plant material, in fact, is usually low and depends on factors such as light, temperature, and soil composition, which can vary with each season [5, 6, 7]. Intriguingly, it has been demonstrated that the cellular plant material (i.e., calluses) obtained from the in vitro culture of fruit pulp contains higher amounts of secondary metabolites, such as PTAs, than the starting material [6, 8]. Fraternale et al. developed a technique to obtain in vitro calluses using explants of the mature pulp of the MRM. Interestingly, the chemical analysis of these calluses revealed that the secondary metabolite content in the calluses obtained from MRM was higher than in the calluses obtained from a common Golden Delicious apple [9]. Furthermore, for both the apple varieties, the content in PTAs in the calluses was higher than the starting plant material [2, 9]. Several types of extraction were performed on the calluses, and the biological activity of the extracts was tested. The aqueous extract of MRM callus showed antiproliferative and antitumorigenic potential, together with chemopreventive properties [10]. Potenza et al. showed that the ethanolic extract of MRM callus (MRME) has antioxidant properties against free radicals, it reduces ROS production in a cell model of H_2_O_2_‐induced oxidative stress, and it has an anti‐inflammatory activity on RAW 264.7 cells treated with LPS [11]. Gubitosa et al. validated in vitro, ex vivo, and in vivo the biological effect of the ethanolic extract: All experimental tests confirmed that MRME protects cells, tissues, or whole organisms from oxidative stress‐induced damage [2]. Finally, Benayada et al. evaluated the in vitro anti‐inflammatory activity of MRME in high glucose and senescence conditions. They demonstrated that the treatment of senescent human umbilical vein endothelial cells (sHUVECs) with 1‐µg/mL MRME significantly reduces the expression of IL‐1β and IL‐8 as compared to untreated sHUVECs, and it upregulates two microRNAs related to senescence regulation and endothelial function, miR‐17 and miR‐126, in the same conditions [6]. Furthermore, they showed that MRME downregulates some inflammatory markers in U937 cells exposed to high glucose and/or lipopolysaccharide (LPS) [6]. These results suggest MRME as a possible inhibitor of senescence‐associated inflammation and indicate a role of its high PTAs content in this biological activity [6]. The transcriptomic and epigenomic reprogramming potential of PTAs has been demonstrated in different cellular models. The activity seems to be associated with changes in the DNA methylation profile of cells treated with single PTAs molecules or with extracts enriched by PTAs [12, 13, 14, 15, 16, 17, 18]. Inflammaging, the chronic, sterile, low‐grade inflammation that occurs during aging, is a well‐recognized factor contributing to the pathogenesis of age‐related diseases (ARDs), such as dementia and cardiovascular diseases [19]. The prevalence of ARDs is rising exponentially due to the growth of the older population and the worsening of lifestyle habits. Aging is characterized by the accumulation of senescent cells (SCs), which are characterized by a state of permanent proliferative arrest; however, they remain metabolically active and acquire a proinflammatory phenotype known as senescence‐associated secretory phenotype (SASP), fueling inflammaging [20]. Physical activity and nutrition play a key role in healthy aging, preventing the onset of ARDs [21, 22]. In particular, consumption of bioactive natural compounds contained in fruit, vegetables, and plants, such as polyphenols, carotenoids, and triterpenic acids, has garnered increasing interest for their potential role in promoting longevity [23]. Thus, given that (i) MRM has been demonstrated to contain bioactive molecules, (ii) the extract obtained from the callus of MRM has been hypothesized to act as a potential inhibitor of senescence‐associated inflammation, and (iii) its transcriptomic and epigenetic effect has never been investigated before, the present study aims to evaluate the nutrigenomic effect of MRME using an in vitro model of cellular senescence.

## 2 Materials and Methods

### 2.1 Mela Rosa Marchigiana Callus and Extract Preparation

The MRM callus used in this study was obtained following the method described by Verardo et al. [9], and MRM callus extract was prepared as described by Gubitosa et al. [2].

### 2.2 Cell Culture, Senescence Characterization, and Viability Assay

HUVEC cells were cultured under standard conditions until replicative senescence and classified as either young (yHUVECs) or senescent (sHUVECs) based on passage number, SA‐β‐galactosidase activity, and p16^INK4a^ expression [24, 25]. The effect of MRME on sHUVEC viability was evaluated using the MTT assay. Detailed methods for cell culture, characterization, and viability assays are provided in the Supplementary Materials.

### 2.3 MRME Treatment Conditions

sHUVECs were treated with MRME at a concentration of 1 µg/mL for 72 h. Untreated sHUVECs (negative control) and yHUVECs were included in the study. Experiments were conducted in triplicate. The selection of the 1‐µg/mL concentration was based on cell viability >80% compared to that of the vehicle‐treated cells and in accordance with the findings of Benayada et al. [6]. After 72‐h incubation, cells were collected and stored at −80°C for subsequent analysis.

### 2.4 DNA and RNA Extraction

Genomic DNA and total RNA were isolated from yHUVECs, sHUVECs, and sHUVECs treated with MRME (sHUVECs MRME) using the RNA/DNA purification kit (Norgen, Thorold, ON, Canada; cat 48700), quantified by Qubit dsDNA HS Assay Kit and Qubit HS RNA assay kit (Thermo Fisher Scientific, Waltham, MA, USA), and stored at −80°C. After purification, RNA integrity was evaluated by separating 1 µg of total RNA by agarose gel electrophoresis (AGE).

### 2.5 RNA Sequencing and Analysis

Total RNA extracted from yHUVECs, sHUVECs, and sHUVECs MRME was used for transcriptomic analysis via RNA‐seq (StarSEQ GmbH, Mainz, Germany). RNA quality and quantity were further evaluated using Agilent 2100 Bionalyzer (Agilent Technologies, Santa Clara, CA, USA). mRNA was isolated, fragmented, and reverse transcribed into cDNA, which was end‐repaired, adapter‐ligated, and PCR‐enriched. Libraries were assessed using the Qubit Fluorometer (Thermo Fisher Scientific, Waltham, MA, USA) and Agilent 2100 Bionalyzer (Agilent Technologies, Santa Clara, USA). Sequencing was performed on the Illumina NextSeq 2000 platform using XLeap SBS chemistry, generating 150 bp paired‐end (PE) reads. Each sample was sequenced to a depth of approximately 25 million PE reads (2×12.5 million reads per sample, 2×150 bp). Reads were trimmed and aligned to the human genome hg38 using the Illumina DRAGEN pipeline. Gene expression was quantified with Salmon, and differential expression was analyzed by edgeR. Low‐expressed genes (≤2 CPM) were filtered out. Normalization was done using the trimmed mean of *M*‐values (TMM). A multidimensional scaling (MDS) plot was generated using the plotMDS function from edgeR with default parameters. Genes were identified as differentially expressed (DEGs) when the following criteria were met: false discovery rate (FDR) ≤ 0.05 and fold change (FC) ≥ 2 or ≤ −2. For the comparison of sHUVECs versus sHUVECs MRME, DEGs were filtered for a FC ≥1.5 or ≤−1.5 in consequence of the lower changes monitored. Where not specified, all plots were generated using the ggplot2 R package.

### 2.6 Pathway Analysis

Pathway analysis was conducted using iPathwayGuide software (Advaita Bioinformatics, United States) to identify the biological pathways associated with the differentially expressed genes (DEGs) observed in the three experimental conditions. The Molecular Signatures Database (MSigDB) gene set was used, and pathways with FDR below 0.01 were considered significantly enriched.

### 2.7 DNA Methylation Analysis

Bisulfite pyrosequencing was performed to evaluate DNA methylation levels in the promoter regions of 12 genes, which were differentially expressed in the comparison between sHUVEC and sHUVEC MRME (Table S1 of Supplementary Materials). To obtain a broader, unbiased, genome‐wide assessment of DNA methylation changes, reduced representation methylation sequencing (RRMS) was also conducted using PromethION 24 (Oxford Nanopore Technology, UK) on DNA from three biological replicates of sHUVEC and sHUVEC MRME. Detailed materials and methods for both pyrosequencing and nanopore RRMS analysis are provided in the Supplementary Materials.

## 3 RESULTS

### 3.1 Differences in the Gene Expression Profile of sHUVECs and yHUVECs

We first decided to investigate the effects induced by senescence on the gene expression profile of HUVEC cells. HUVECs were defined as follows: (i) young HUVECs (yHUVECs), characterized by a replicative passage number <6, (SA)‐β‐galactosidase activity <20%, and markedly reduced p16^INK4a expression relative to senescent counterparts; (ii) senescent HUVECs (sHUVECs), defined by a replicative passage number >15, (SA)‐β‐galactosidase activity >60%, and significantly elevated p16^INK4a expression compared with yHUVECs, as reported previously [6].

**FIGURE 1:**
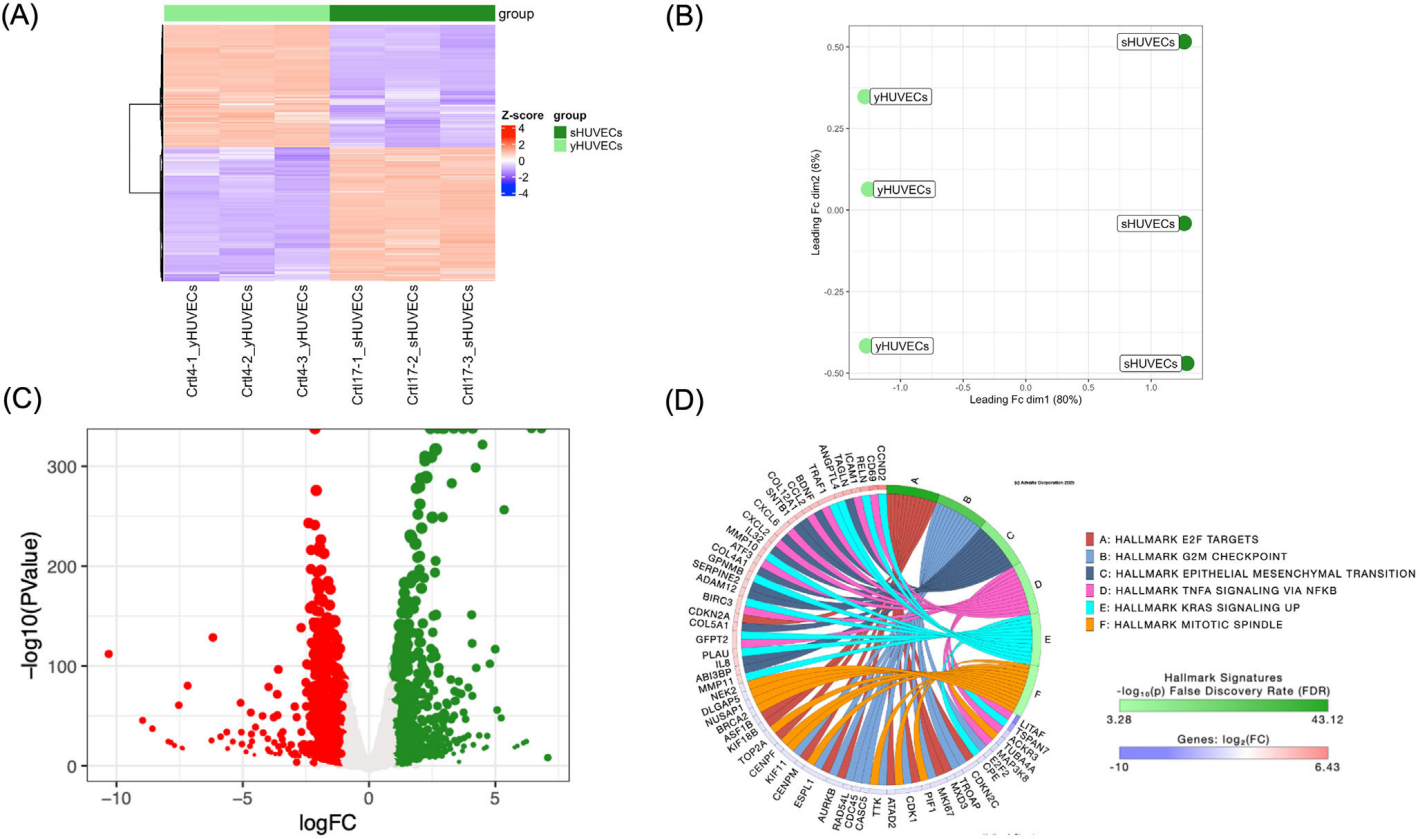
Genome‐wide analysis of the gene expression modulations induced by senescence in HUVEC cells. HUVEC cells were subjected to passage‐induced senescence and analyzed by RNA‐seq using the R package edgeR with a likelihood ratio test (LRT); the resulting *p* values were adjusted using the Benjamini–Hochberg (BH) method. All experiments have been conducted using three biological replicates for each condition. (A) Hierarchical clustering heatmap of all DEGs (FDR ≤ 0.05) of yHUVECs and sHUVECs. As reported in the color key, positive *Z*‐scores (red) are associated with higher expression values, while negative *Z*‐scores (blue) indicate a lower expression value. (B) Multidimensional scaling (MDS) analysis comparing yHUVECs and sHUVECs. (C) Volcano plot of DEGs obtained comparing yHUVECs and sHUVECs. Significantly downregulated genes in sHUVECs are shown in red, while upregulated genes are shown in green. (D) Circos plot showing the pathways found modulated using the Molecular Signatures Database (MSigDB) Hallmark signatures, and relative DEGs were obtained comparing yHUVECs and sHUVECs. Pathways were considered significantly enriched when *p* < 0.01 and are reported in figure.

**FIGURE 2:**
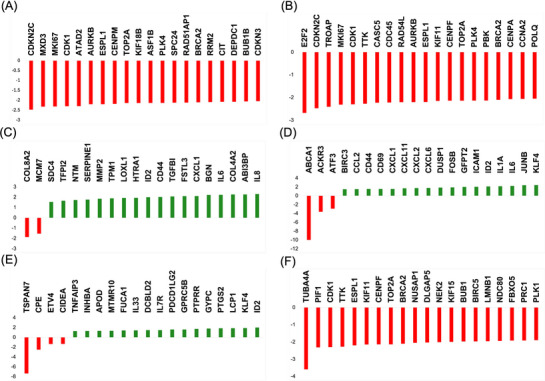
Quantitative transcript variations of the DEGs belonging to the six pathways modulated by senescence in HUVEC cells. The first 20 differentially expressed genes (DEGs) enriching the six pathways found modulated by senescence in HUVEC cells are shown as green bars (upregulated DEGs) or red bars (downregulated DEGs). (A) E2F targets pathway; (B) G2M checkpoint pathway; (C) epithelial mesenchymal transition pathway; (D) TNF‐α signaling via NFKB pathway; (E) KRAS signaling up pathway; (F) mitotic spindle pathway. Data on the *y* axes are reported as the logarithm of the fold change (logFC).

RNA‐seq was conducted using three biological replicates for yHUVECs and sHUVECs. The overall similarity between samples has been assessed through either a hierarchical clustering (Euclidian distance) (Figure 1A) and a MDS analysis (Figure 1B), allowing the clustering of the triplicates into distinct groups in function of culture passages, suggesting that senescence had a high impact on gene expression. MDS shows that dimension 1 (dim1), which explains 80% of the total variability of our dataset, is driven by senescence, while dim2 (6%) variation seems to be related to intragroup variability. Differential enrichment analysis by edgeR package allowed us to identify genes affected by senescence in HUVECs. According to results, a total of 1452 DEGs were identified in sHUVECs compared to yHUVECs (Table S2 of Supplementary Materials), with 759 upregulated and 693 downregulated genes in sHUVEC (Figure 1C). The DEGs were analyzed for pathway enrichment using the iPathwayGuide software. According to results, we found six pathways as significantly enriched (Figure 1D): E2F targets (*p* < 0.01), G2M checkpoint (*p* < 0.01), epithelial mesenchymal transition (*p* < 0.01), TNFα signaling via NFKB (*p* < 0.01), KRAS signaling up (*p* < 0.01), and mitotic spindle (*p* < 0.01). “E2F Targets” pathway included 100 DEGs, which were downregulated and only two DEGs which were upregulated in sHUVECs compared to yHUVECs, while “G2M checkpoint” included 84 genes, which were significantly downregulated and one gene which was upregulated, indicating the downregulation of both pathways. “Epithelial mesenchymal transition” pathway involved 57 upregulated genes and five downregulated genes, “TNFα signaling via NFKB” pathway included 48 upregulated genes and three downregulated genes, while “KRAS signaling up” included 36 upregulated genes and seven downregulated genes, suggesting, in this case, the upregulation of these three pathways. Finally, “Mitotic spindle” included 38 downregulated and only four upregulated genes. The first 20 DEGs enriching the six pathways are shown in Figure 2.

### 3.2 Modulation of the Gene Expression Profile Induced by MRME in sHUVEC

**FIGURE 3:**
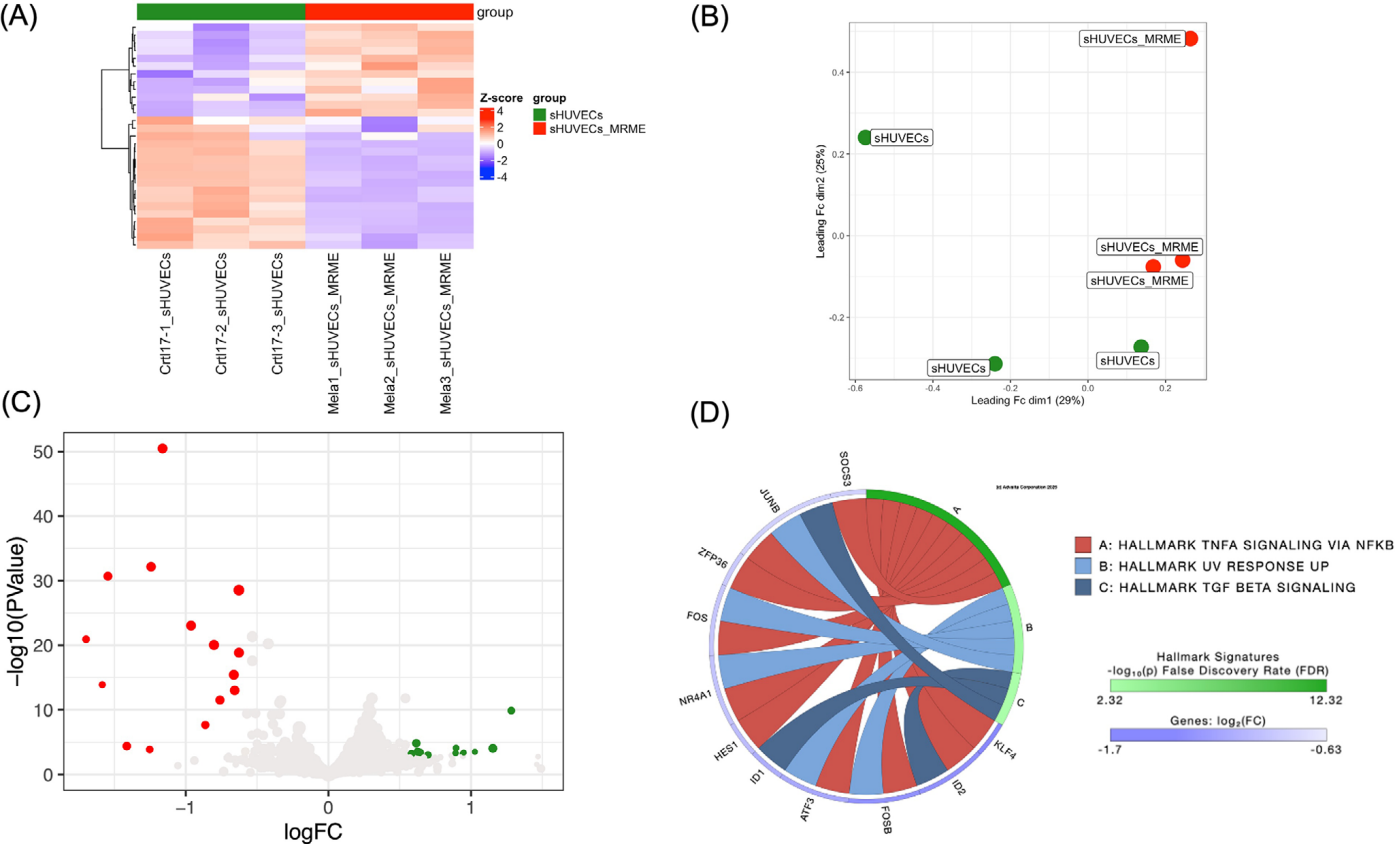
Genome‐wide analysis of the gene expression modulations induced by MRME treatments in senescent HUVECs (sHUVECs). HUVECs were treated with an MRME at a concentration of 1 µg/mL for 72 h and analyzed by RNA‐seq using the R package edgeR with a likelihood ratio test (LRT); the resulting *p* values were adjusted using the Benjamini–Hochberg (BH) method. All experiments have been conducted using three biological replicates for each condition. (A) Hierarchical clustering heatmap of all DEGs (FDR ≤ 0.05) of untreated and MRME‐treated sHUVECs. As reported in the color key, positive *Z*‐scores (red) are associated with higher expression values, while negative *Z*‐scores (blue) indicate a lower expression value. (B) Multidimensional scaling (MDS) analysis comparing sHUVECs and sHUVECs MRME. (C) Volcano plot of DEGs obtained comparing sHUVECs and sHUVECs MRME. Significantly upregulated genes in sHUVEC MRME are shown in green; downregulated genes are shown in red. (D) Circos plot showing the pathways found modulated using the Molecular Signatures Database (MSigDB) Hallmark signatures and relative DEGs obtained comparing sHUVECs and sHUVECs MRME. Pathways were considered significantly enriched when *p* < 0.01 and are reported in the figure.

**FIGURE 4:**
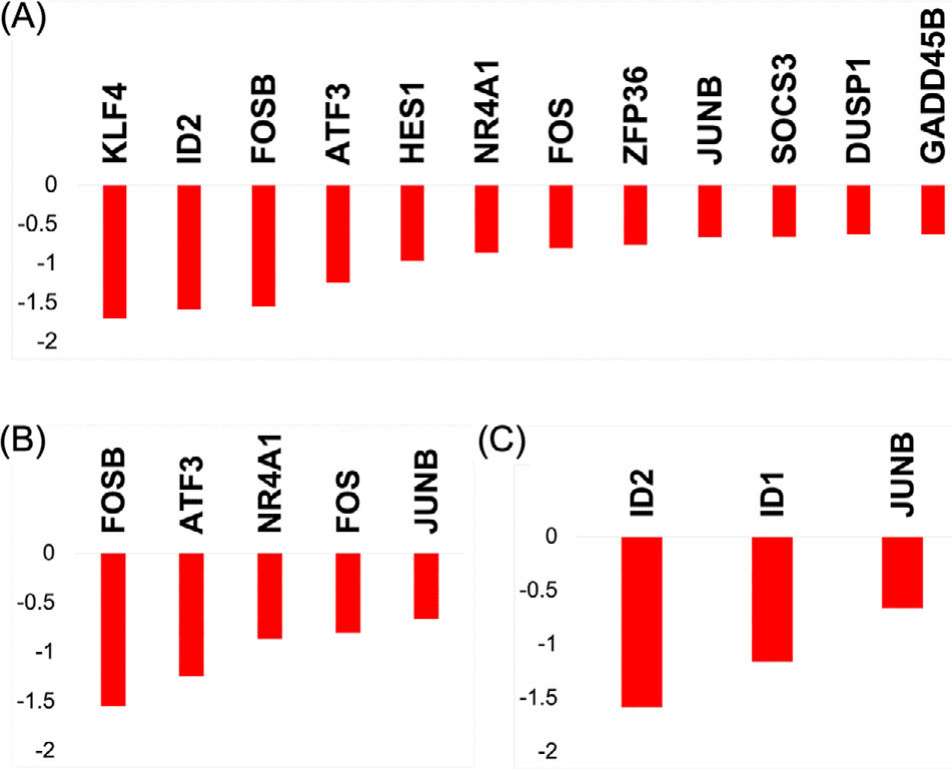
Quantitative transcript variations of the DEGs belonging to the three pathways modulated by MRME in sHUVECs. DEGs enriching the pathways found modulated by MRME treatments in sHUVEC cells are shown as green bars (upregulated DEGs in sHUVEC MRME) or red bars (downregulated DEGs). (A) TNF‐α signaling via NFKB pathway; (B) UV response pathway; (C) TGF‐β signaling pathway. Data on the *y* axes are reported as the logarithm of the fold change (logFC).

First of all, the effect of MRME on sHUVECs viability has been evaluated by treating cells with different concentrations of MRME (ranging from 0.76 to 12.5 µg/mL) for 72 h. MRME treatments did not show any significant effect on sHUVEC cell viability (Figure S1 of Supplementary Materials). RNA‐seq was then conducted on three biological replicates of sHUVECs treated for 72 h with MRME at the concentration of 1 µg/mL and compared to untreated sHUVECs to evaluate the impact of MRME exposure on the gene expression of sHUVECs. This condition was chosen in consequence of previous works on MRME extracts [6]. Hierarchical clustering (Euclidian distance) was performed, showing the clustering of the triplicates into distinct groups (Figure 3A). Regarding MDS analysis, even if only slight differences are present among the two groups, the samples can be anyway clustered matching both dim1 (29%) and dim2 (25%) components (Figure 3B). We found a total of 26 DEGs in sHUVECs MRME compared to untreated sHUVECs, with 11 upregulated and 15 downregulated genes (Figure 3C). The list of DEGs is reported in Table S3 of Supplementary Materials. Interestingly, iPathwayGuide analysis of DEGs showed that three pathways were significantly enriched by MRME treatments: TNFα signaling via NFKB (p < 0.01), UV response up (p < 0.01), and TGF‐β signaling (*p* < 0.01) (Figure 3D). “TNFα signaling via NFKB” included 12 DEGs (Figure 4A): ID2, GADD45B, JUNB, HES1, ATF3 NR4A1, KLF4, FOS, FOSB, ZFP36, DUSP1, and SOCS3. All these genes were significantly downregulated by MRME treatments in sHUVECs. The “UV response up” pathway included five DEGs, which were downregulated in sHUVECs treated with the extract (Figure 4B): JUNB, ATF3, NR4A1, FOS, and FOSB. Finally, TGF‐β signaling pathway included three genes as follows (Figure 4C): ID1, ID2, and JUNB that were, even in this case, downregulated in sHUVECs MRME.

### 3.3 Effect of MRME on DNA Methylation in sHUVEC

**FIGURE 5:**
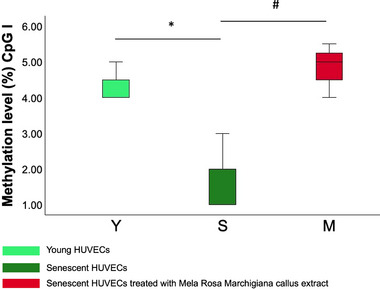
Methylation level of the first CpG site on the selected area of the SOCS3 promoter. Methylation level measured by pyrosequencing analysis in the I CpG site of the selected area of the SOCS3 promoter in the three groups: Y (young HUVECs), S (senescent HUVECs), M (senescent HUVECs treated with MRME). The experiment was performed using three biological replicates for each condition and two technical replicates for the analysis. There was a statistically significant increase in yHUVECs compared to sHUVECs (*, *p* < 0.05) and in sHUVECs MRME compared sHUVECs (#, *p* < 0.05). The boxes represent the interquartile range (25th–75th percentile), the line inside the box indicates the median, and the whiskers extend to the smallest and largest values within 1.5 times the interquartile range. ANOVA test or Kruskall–Wallis test was used to compare differences in methylation levels between groups.

To explore whether the observed transcriptional changes were associated with DNA methylation, we performed pyrosequencing analysis in the promoter regions of the 12 DEGs enriched in the TNFα signaling via NFKB pathway. No significant differences in mean promoter methylation levels were observed across conditions (Figure S2). However, methylation at a specific CpG site within the SOCS3 promoter was significantly altered by MRME (Figure 5). Along the same line, complementary genome‐wide DNA methylation profiling using PromethION24 (Oxford Nanopore technology, UK) also showed no significant MRME‐induced changes in CpG methylation at either single‐site (data not shown) or promoter levels (Figure S3). Detailed results are provided in the Supplementary Materials.

## 4 DISCUSSION

Several dietary components have been suggested to affect the genome by regulating the gene expression profile of cells through epigenetic and not‐epigenetic mechanisms [26, 27, 28]. Among these, bioactive molecules contained in fruit and vegetables have been demonstrated to exert an active role in promoting human health through a nutrigenomic effect [28]. In particular, polyphenols, carotenoid, and terpenoids have gained increasing attention for their potential role in promoting longevity [23]. Here, we focused attention on the effects mediated by an ethanolic extract of the callus of Mela Rosa Marchigiana (MRME), an ancient Italian apple containing PTAs [9] secondary metabolites present in small amounts in fruit and vegetables, with known beneficial effects for human health [29, 30, 31, 32]. Its biological effect has been previously investigated using different models, with findings suggesting anti‐inflammatory properties and potential anti‐aging activity of MRME [2, 6]. Although MRM callus is a good source of active compounds, the transcriptomic and epigenetic effects of its extract have never been studied before. This research aimed to investigate the impact of MRME on global gene expression and on DNA methylation using an in vitro model of replicative senescence. First, we focused our attention on the differences between yHUVECs and sHUVECs to identify the transcriptomic variations associated with the senescent phenotype. According to our results, a list of 1452 DEGs was identified, with 759 genes upregulated and 693 genes downregulated. Gene set enrichment analysis identified six significantly enriched biological pathways in SCs: E2F targets, G2M checkpoint, epithelial–mesenchymal transition (EMT), TNFα signaling via NFKB, KRAS signaling up, and mitotic spindle. E2F is known to play a major role in cell cycle regulation and its targets include genes regulating cell cycle, cell proliferation, and apoptosis [33]; similarly, the G2M checkpoint is a critical stage in cell cycle progression, preventing cells from entering mitosis when DNA is damaged [34]. Both were downregulated in sHUVECs compared to yHUVECs. This result is not surprising, since the decreased expression levels of cell cycle genes are typical of senescence and are in line with current literature [35]. EMT pathway is responsible for the acquisition of the mesenchymal phenotype by epithelial cells [36]. It is a key regulator of cancer development, favoring metastasis formation, and it has been suggested to be activated by cellular senescence [37]. In fact, the inflammatory environment characterizing SASP has been demonstrated to drive EMT [37]. Even though this pathway is peculiar to epithelial cells, also endothelial cells have been shown to transit into a mesenchymal phenotype throughout the activation of the endothelial to mesenchymal transition pathway (EndMT) occurring during aging [38, 39]. The upregulation of DEGs involved in the EMT pathway in sHUVECs suggests an activation in replicative SCs, possibly due to the inflammatory status characterizing SCs. Other enriched pathways were TNFα signaling via NFKB pathway, which regulates inflammation; KRAS signaling pathway, which regulates signal transduction; and mitotic spindle pathway, which is implicated in the mitosis process. TNFα via NFKB is a well‐known pathway regulating inflammation [40], and its involvement in aging has been extensively studied. NFKB signaling was suggested to increase with senescence, and its activity has been associated with numerous ARDs [41, 42]. Furthermore, increasing literature suggests that NFKB signaling promotes aging, enhancing the manifestation of SASP [41, 43]. KRAS signaling stimulates cell growth, cell division, cell survival, and regulates cell death [44]. Its upregulation, although paradoxical, could be part of the molecular events associated with the induction of replicative senescence. Activation of a DNA damage response was found to induce senescence in response to overexpression of oncogenic RAS [45]. Finally, the mitotic spindle pathway is downregulated in our experimental condition, in line with the reduction of the mitotic process during senescence. Ohori et al. performed RNA‐seq on HUVECs at different culture times to study transcriptome changes during senescence, identifying alterations in pathways related to cell cycle, DNA repair, metabolism, and chromosome organization [46]. Notably, G2M checkpoint and mitotic spindle processes were significantly impacted, consistent with our findings [46]. Ramini et al. analyzed HUVECs rendered senescent by high glucose, revealing enrichment of interferon alpha/beta signaling pathway [47]. They also assessed IL‐6, IL‐1β, and IL‐8 expression in yHUVECs (CPD = 15) and sHUVECs (CPD = 35) under standard glucose conditions, reporting changes in IL‐6 and IL‐8 levels consistent with our results (see Table S2 of Supplementary Materials). Similar results have also been described in another study [48], which also showed Dnmt1 downregulation, consistent with our data (see Table S2 of Supplementary Materials). After having analyzed the differences in gene expression between sHUVECs and yHUVECs, we focused on the effects induced by MRME on sHUVECs. The results suggest an interesting nutrigenomic effect of MRME, highlighting its role in modulating gene expression. Notably, according to transcriptomic analysis results, 26 genes were differentially expressed comparing sHUVECs MRME and sHUVECs. It remains unclear whether the extract's effects arise from the cumulative action of all its multiple constituents or from specific polypharmacological compounds. Increasing attention is being directed toward phytocomplexes rather than isolated secondary metabolites, as the diverse chemical components of a plant can act synergistically to yield a more potent and balanced therapeutic effect [49, 50, 51].

**FIGURE 6:**
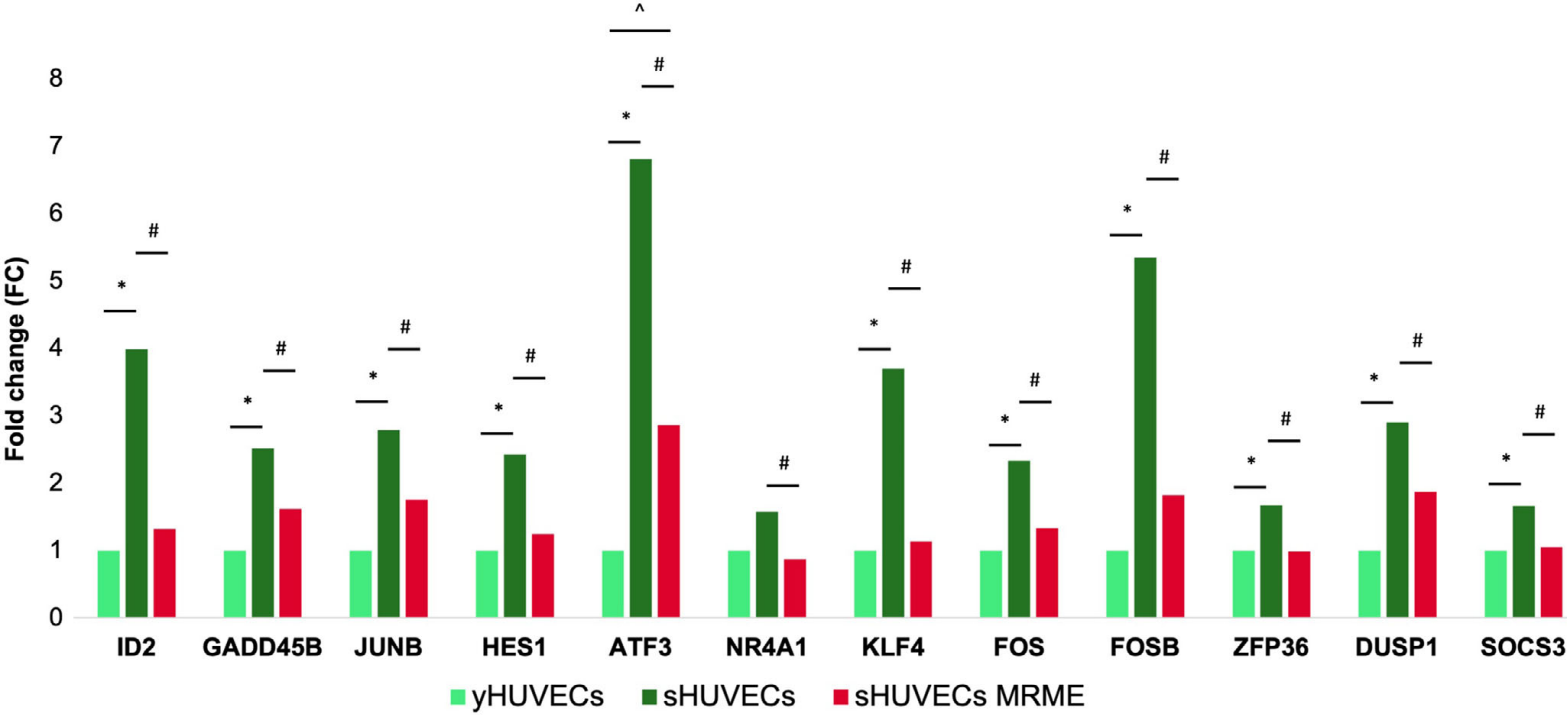
Expression levels of TNF‐α pathway genes modulated by MRME. The relative expression levels of the 12 TNF‐α pathway genes in the three conditions as follows: yHUVECs, sHUVECs, and sHUVECs MRME. *p < 0.05 yHUVECs versus sHUVECs; #*p* < 0.05 sHUVECs versus sHUVECs MRME; ^*p* < 0.05 yHUVECs versus sHUVECs MRME.

Indeed, the contribution of individual MRME phytocompounds (e.g., PTAs) to gene regulation remains only partially understood, with some compounds still uncharacterized and others reported to exert modulatory effects [52, 53, 54, 55]. In particular, Oleanolic acid was demonstrated to modulate gene expression in HUVECs [53, 56]. Pathway analysis was conducted to determine whether the DEGs obtained comparing sHUVECs MRME and sHUVECs were enriched in specific biological pathways. According to results, MRME significantly modulated three main pathways as follows: TNFα signaling via NFKB pathway, UV response up pathway, and TGF‐β signaling. Interestingly, TNFα signaling via NFKB pathway was the most significantly modulated pathway in sHUVECs MRME with respect to sHUVECs. Since the inhibition of NFKB activation has been suggested to delay or revert the aging phenotype [42], the observed downregulation of TNFα/NFKB‐related genes suggests that MRME may suppress pro‐inflammaging processes, driving the senescence phenotype. In line with this evidence, MRME could be potentially proposed not only as an anti‐inflammatory agent but also as a possible modulator of aging. This hypothesis is consistent with previous studies reporting potential anti‐aging effects of PTAs in various experimental models [57, 58, 59, 60, 61, 62]. However, further comparative in vivo studies, using the phytocomplex, are necessary to confirm the anti‐aging activity to better elucidate their potential clinical application. Previous literature showed that MRME downregulated the expression of inflammatory markers and upregulated miRNAs involved in inflammation and in the inhibition of cellular senescence [6], in line with our findings [6]. In addition to TNFα via NFKB pathway, MRME also modulated other pathways, including the UV response pathway, a process activated in response to ultraviolet radiation [63], and TGF‐β signaling pathway, which regulates various biological mechanisms, such as cell proliferation, cell cycle regulation, DNA damage response, telomere regulation, and autophagy [64]. Increasing literature suggests that TGF‐β signaling is implicated in the aging process and in the occurrence of several age‐associated diseases [65]. Interestingly, the inhibition of TGF‐β signaling has been suggested to retard cellular senescence in human pluripotent stem cells derived endothelial cells [66]. Although only three DEGs in this pathway were downregulated by MRME, these changes indicate its impact on senescence‐related mechanisms. After having identified pathways modulated by the MRME and by senescence, we concentrated our attention on the expression trends of individual genes, focusing on the 12 genes downregulated by MRME in sHUVEC that are part of the TNFα signaling via NFKB pathway. The aim of this observation was to specifically assess whether the expression levels of these genes changed with senescence and if the treatment of sHUVECs with MRME was able to restore the expression level to those occurring in young cells. Interestingly, we found that the expression levels of most of these 12 genes increased in sHUVECs with respect to yHUVECs and were lowered by MRME treatments of sHUVECs, suggesting that the senescence‐associated upregulation of these genes can be attenuated by MRME exposure (Figure 6). Among the 12 genes analyzed, transcriptional regulators and factors involved in cytokine response were identified, including FOS, FOSB, and JUNB‐components of the AP‐1 complex linked to inflammation [67]. Karakaslar et al. showed JUN and FOS activation as markers of immune aging in mice [67], consistent with our findings of increased JUN, JUNB, and FOS expression in senescent HUVECs. A secondary aim of this study was to verify whether the alterations of gene expression levels found in the comparison between sHUVECs MRME and sHUVECs were due to epigenetic alterations in DNA methylation. We focused on the 12 DEGs that were involved in the TNFα via NFKB pathway, and we analyzed the DNA methylation levels in selected areas of the promoters of these genes by pyrosequencing. Results revealed no significant differences in mean methylation levels at promoter regions of the 12 genes involved in the TNFα signaling pathway between yHUVECs, sHUVECs, and sHUVECs MRME. Only the first CpG site in the selected area of the SOCS3 promoter showed a significantly different level of methylation in sHUVECs compared to yHUVECs and in sHUVECs MRME compared to sHUVECs, even though the difference observed at this single CpG site is small and close to the lower range of reliable detection by pyrosequencing (as indicated by the low methylation percentages). Therefore, it should be interpreted with caution and may reflect technical or biological variability rather than a consistent epigenetic modification.

Similarly, genome‐wide DNA methylation analysis performed using nanopore sequencing platform also revealed no significant MRME‐induced quantitative changes in CpG methylation at either single‐site or promoter level in our experimental setting. Thus, although we did not sequence 100% of the genome but only regions highly enriched in CpGs, it is unlikely that the gene expression changes induced by MRME are primarily driven by major DNA methylation variations caused by the extract in this experimental setting. In line with this observation, we found that the expression level of DNA methyltransferase enzymes DNMT1, DNMT3A, and DNMT3B were not influenced by MRME treatments, although DNMT1 and DNMT3B were downregulated in sHUVECs as compared to yHUVEC. This result is in agreement with the present literature and with the global hypomethylation occurring during senescence [68]. This study has limitations that need to be addressed. First, it was conducted using three biological replicates per group. Although the number of replicates is relatively small and may not fully capture the underlying variability, it represents a reasonable compromise for an exploratory investigation aimed at generating a preliminary dataset. This design enabled us to capture initial molecular insights that can be further tested and expanded in future studies.

Second, since we did not apply ATAC (chromatin accessibility) or HiC (topological association domain) sequencing, nor assays addressing histone post‐translational modifications (PTMs), our results may not capture the full complexity of 3D chromatin (re)organization upon senescence, an aspect that warrants further exploration [69, 70, 71].

Third, in line with the exploratory aim of this study, our analysis focused exclusively on the expression level of genes without measuring protein levels, enzymatic activities, or actual pathway activation, only allowing us to identify preliminary molecular trends in mode of action.

Furthermore, to estimate the clinical relevance of our findings, our results need further validation in more complex biological systems to strengthen their translatability. Nevertheless, this study sheds light, for the first time, on the nutrigenomic effect of MRME, corroborating its interesting anti‐inflammatory potential already documented in previous works. Further studies will be necessary to elucidate the mechanisms responsible for the modulation of gene expression induced by MRME and to fully understand if this extract could serve as a potential agent for promoting overall health, as well as its potential applications in specific clinical contexts.

## Conflicts of Interest

The Authors declare no conflicts of interests.

## Supporting information




**Supporting Information File 1**: mnfr70336‐sup‐0001‐SuppMat.docx.

**Supporting Information File 2**: mnfr70336‐sup‐0001‐SuppMat.docx.

## Data Availability

No data are available.
