# Supplementary material for: The Nutrigenomic Effect of Mela Rosa Marchigiana Callus Extract on Cellular Senescence: Insight From a Preliminary In Vitro Study
Source: Mol Nutr Food Res. 2025 Nov 22;69(24):e70336. doi: 10.1002/mnfr.70336 (PMC12700053; doi:10.1002/mnfr.70336)

Supplementary Materials

**Supplementary Materials and Methods**

***Supplementary materials and methods: Cell culture, caracterization and cell viability assay***

Cryopreserved Human umbilical vein endothelial cells (HUVECs) (Clonetics, Lonza, Switzerland) were cultured in endothelial growth medium (EGM-2) (Lonza, Switzerland), composed of endothelial basal medium (EBM-2) (Lonza, Switzerland) and the SingleQuot Bullet Kit (Lonza, Switzerland). The cells were seeded at a density of 5,000/cm^2^ in T75 flasks (Corning Costar, Sigma Aldrich, St. Louis, MO, USA), sub-cultured when they reached 70–80% confluence, and maintained in a humidified atmosphere of 5% CO_2_ at 37 °C.

Cells were expanded through multiple passages under these conditions until they reached replicative senescence. The cumulative population doubling (cPD) was determined as the sum of PD changes using the formula: log10(F) – log10(I))/log10(2) ^[24]^, where **F** represents the number of cells at the end of the passage and **I** the number of seeded cells. Cells were classified as either young (y) or senescent (s) based on replicative passages, senescence-associated (SA) β-Galactosidase activity, and p16^INK4a^ expression levels. Specifically, young HUVECs (yHUVECs) were defined as those with a replicative passage number below 6, SA-β-Galactosidase activity below 20%, and significantly lower p16^INK4a^ expression compared to sHUVEC. In contrast, sHUVEC were identified by a replicative passage number exceeding 15, SA-β-Galactosidase activity above 60%, and significantly higher p16^INK4a^ expression than yHUVECs. To evaluate the effect of MRME on cell viability, sHUVECs were grown in 24-well plates at a density of 5,000 cells/cm^2^ and exposed to different doses of MRME (12.5, 6.125, 3.06, 1.57, 0.76 μg/ml) for 72 h. Vehicle (70% ethanol)-treated cells were used as control. Briefly, MTT (5 mg/ml) solution was added to each well (10 μl per every 100 μl) and incubated at 37 °C for 4 h. After the incubation period, the obtained formazan salt was dissolved in dimethyl sulfoxide (DMSO) and the absorbance was measured by a microplate reader (MPT Reader, Invitrogen, Milano, Italy) at the optical density of 570 nm.

***Supplementary Materials and Methods: DNA methylation analysis***

Isolated genomic DNA was converted with sodium bisulfite using the EZ DNA Methylation Gold kit (ZYMO research, Orange, USA; cat D5006) following manufacturer’s instructions. 200 ng of DNA were mixed in PCR tubes with 130 μl of bisulfite mix solution. Samples were incubated in the thermal cycler device (2720 Thermal cycler, Applied Biosystem, Waltham, USA) for 160 min using the following reaction sequence: 98°C for 10 min followed by 150 min min at 64°C. Converted samples were purified using ZYMO spin IC columns and eluted in 20 μL of elution buffer. PCR amplification was performed using PyroMark PCR kit (Qiagen Inc., Venlo, the Netherlands; cat: 978703) in a thermal cycling device (2720 Thermal cycler, Applied Biosystem, Waltham, USA). Primers used to amplify the regions of interests are shown in Table S1 of Supplementary Materials. Briefly, 15 μl of master mix, 3 μl of coral load, 1.2 μl of each primer, 8.6 μl of water, and 1 μl sample (10 ng) were mixed for each reaction and run under the following thermal cycling conditions: 95°C for 15 min, followed by 45 cycles of 30 sec at 94°C, 30 sec at 56°C, and 30 sec at 72°C, followed by a final extension step for 10 min at 72°C. Amplicons were checked for specificity and efficiency of the reaction by gel electrophoresis and then pyrosequenced using the PyroMark Q24 device (Qiagen Inc., Venlo, the Netherlands). PCR products were immobilized on Streptavidin-Sepharose beads (S-GE17-5113-01Streptavidin Sepharose High Performance, Sigma Aldrich) mixing in each well of a 24-well PCR plate 1 μl of Streptavidin-Sepharose beads, 40 μl of Binding Buffer (Qiagen Inc., Venlo, the Netherlands) 30 μl of ultra-distilled water and 9 μl of amplicons from each sample. The mixture was incubated at room temperature with shaking. PyroMark Workstation was prepared using 50 ml 70% ethanol, 40 mL Denaturation Solution (Qiagen Inc., Venlo, the Netherlands), 50 ml Wash Buffer (Qiagen Inc., Venlo, the Netherlands), and 50 ml and 70 ml ultra-distilled water. 0.75 μl of sequencing primer (specific for each sample) and 24.25 μl of Annealing Buffer (Qiagen Inc., Venlo, the Netherlands) were transferred to each well of a PyroMark Q24 plate (Qiagen Inc., Venlo, the Netherlands). The 24-well PCR plate and the Pyromark Q24 plate were transferred to the Workstation. All PCR Sepharose-product complexes were subjected to vacuum filtration and sequentially washed with ethanol, denaturation solution and wash buffer. The vacuum was turned off to allow the transfer of the complexes in the PyroMark Q24 plate. The vacuum filters were washed extensively in ultra-distilled water. The PyroMark Q24 plates were placed into the Pyromark PyroMark Q24 Sequencer (Qiagen Inc., Venlo, the Netherlands) and sequencing reactions were carried out using cartridges containing enzyme, substrate and nucleotides. The degree of methylation was defined as the percentage of methylated cytosines over the sum of methylated and unmethylated cytosines.

Statistical analysis was performed using SPSS (IBM, version 29, USA). Shapiro-wilk method was used to test for normality of data. ANOVA test or Kruskall-Wallis test was used to compare differences in methylation levels between groups.

PromethION 24 (Oxford Nanopore Technologies, UK) was also used to assess the DNA methylation in sHUVECs and sHUVECs MRME. Adaptive sampling mode was applied to selectively enrich for approximately 310 Mb of the human genome, targeting regions highly enriched in CpG sites, including all annotated CpG islands, shores, shelves, and over 90% of promoter regions.

Three biological replicates for each condition for a total of six samples were sequenced using PromethION24 Instrument (Oxford Nanopore Technologies, Oxford, UK) and R10.4.1 flow cells (Oxford Nanopore Technologies, Oxford, UK) together with four additional samples to reach the recommended DNA molarity for optimal sequencing. In total we sequenced ten samples in two flow cells. Briefly, for all the samples, isolated genomic DNA was quantified using Qubit 4 Fluorometer (Thermo Fisher Scientific) and Qubit dsDNA HS kit (Thermofisher, Dilbeek, Belgium). DNA purity was assessed using Little Lunatic (Pleasanton, CA , USA) and integrity was evaluated with the Fragment Analyzer (Agilent HQ, Santa Clara, CA, USA) using the Agilent DNF-464 HS Large Fragment Kit. After quality control, 1-2 ug of DNA (depending on the sample) was fragmented using Megaruptor 3 (Hologic Diagenode, Ougree, Belgium) to obtain fragments having a size of 6-8 kb. Fragment size was confirmed using Fragment Analyzer, using DNF-492 Standard Sensitivity kit. Then, a volume of 50 ul of fragmented and size selected DNA, corresponding to a range between 732 ng and 1500 ng (equivalent to 154-335 fmol, depending on the sample) was used for library preparation. Libraries were prepared following the protocol for Reduced Representation Methylation Sequencing released By Oxford Nanopore on January 2025 [Ligation sequencing gDNA V14 - reduced representation methylation multiplex sequencing (RRMS) (SQK-NBD114.24) V RRMS_9209_v114_revE_07Jan2025]. The Ligation sequencing kit SQK-NBD114.24 (Oxford Nanopore Technologies, Oxford, UK) was used to prepare libraries. Briefly, DNA was repaired and end-prepped using NEBNext FFPE DNA Repair Mix (New England Biolab, Ipswich, MA, USA, cat:#M6630) and NEBNext® Ultra II End Repair / dA-tailing Module (New England Biolab, Ipswich, MA, USA, cat: #E7546). Native Barcoding ligation was performed using the NEB Blunt/TA Ligase Master Mix (New England Biolab, Ipswich, MA, USA, cat: #M0367), assigning a unique barcode to each of the 10 samples included in the experiment. Barcoded samples were pooled, and sequencing adapters were ligated to the pooled DNA using the NEBNext Quick Ligation Module (New England Biolab, Ipswich, MA, USA, cat: #E6056). The final multiplexed library was purified, eluted in Elution Buffer and loaded in two flow cells which were sequenced simultaneously on PromethION24 Instrument (Oxford Nanopore Technologies, Oxford, UK). The sequencing run was carried out for 96 hours, after 24 hours and 48 hours of sequencing both the flow cells were flushed, and a fresh library was loaded.

The two flow cells respectively produced an output of 78.46 M and 79.25 M reads, with an estimated N50 of 2.83 kb for the first flow cell and 1.07 kb for the second, resulting in a total base output of 92.63 Gb and 89.9 Gb . Basecalling and methylation calling of the Nanopore data was performed on the instrument by MinKNOW using dorado 7.6.8 (High-accuracy model v4.3.0, 400 bps). The reads were aligned to the hg38 genome reference using minimap2 (<https://academic.oup.com/bioinformatics/article/34/18/3094/4994778?login=true>) and bedMethyl files were generated per sample using modkit. The edgeR package was used to perform differentially methylation analysis at CpGs loci and Promoter regions following the workflow for Bisulfite Sequencing differentially methylation analysis (section 4.8 in the EdgeR user’s guide).

**Table S1**. Primers used for bisulfite pyrosequencing analysis.

| Gene name | Primer forward | Primer reverse | Primer seq |
| --- | --- | --- | --- |
| **ID2** | 5'-GGT TTG GGA GAG TTT GGG ATT G-3' | 5'-Biotin-AAACCC CCAAACAAACTCTCT AAC-3' | 5'-GGG TTTAGTAGG TAT TGATTA GT-3' |
| **GADD45B** | 5-AGG AGG GGAGAATGA T TAT TGA G-3' | 5'-Biotin-CCAAAA CCT TCC CAC AAAACT AAA-3' | 5’-GGG AGAATG AT TATTGAGG T-3' |
| **JUNB** | 5'-ATT TGT T AGT GGA T TAG GGA AAT TA-3' | 5’-Biotin-CAA CTCCCAACT CCC TAC TA C-3' | 5’-GGT TAGTGGTAGTTGTIT ATA AGG-3' |
| **HES1** | 5-AGAGGGAGAGTAGTAAAG GGT TAAAA-3' | 5-Biotin-CAA ACT TC T CCC ACAATA ACT TC AA-3' | 5'-AGTAGTAAAGGG TTAAAATTT-3' |
| **ATF3** | 5'-GGTTTGGGT ATT ATT GGT TATGT-3' | 5' Biotin-CCAACC C CT CTC TCTCCA T-3' | 5'-GTA TTATTG GTT ATG TTT GGA A-3' |
| **NR4A1** | 5-GTT TAGTGG GTTTGG GAG TT-3' | 5'-Biotin-TCT TCT ATACAC TCC CCC AAA TTT-3' | 5-'GAG GAG TIT AT TAT AGA TT-3' |
| **KLF4** | 5-GGG AGG GGG AGGGGA AG A-3' | 5'-Biotin-CCCCCTAC CCC AC AAT CTT C-3' | 5'-GGTAGTGAAGTT AGT TAG GTAGTT G-3' |
| **FOS** | 5-AGTTITGGG GGG AGT TAT T-3' | 5'-Biotin-CAA CA CTA CT TA TAA CAA-3' | 5'-AGG AGGGGGTAG GGA-3' |
| **FOSB** | 5'-GGT TTAGAGGGT TAT GTAAGT GAT TAGAT-3' | 5-Biotin-ACAAAC C CCAACC TAT ATTAT ATA CCC-3' | 5-'AGATITTTTGTTGGATAGTG-3' |
| **ZFP36** | 5-GGG GAT TA T AGT AGATGGTAG TTAGA-3' | 5'-Biotin-CCA ATC CAAAAC CAACCAAAC TAC-3' | 5'-GAG AGT AGGTTATTT ATAGGAAAT T-3' |
| **DUSP1** | 5'-GGGGTG T GTT TGA ATG GTGATT A-3' | 5-Biotin-TCCTCC TC CCC CTA ACCTA ARA C-3' | 5'-ATTAGTTTAGGAGTTGGG T-3' |
| **SOCS3** | 5'-GGT AGG TTTTTT TGT AAT GTT TAG TTAT-3' | 5'-Biotin-CTC TACCAAAAATCAACCT T CTTAAAA-3' | 5'-AGG GTG AGG GAG GGG-3' |

**Supplementary Results**

**Table S2.** Transcriptomic analysis results: list of differentially expressed genes (DEGs) obtained comparing young HUVECs (yHUVECs) and senescent HUVECs (sHUVECs). Genes were identified as DEGs when the following criteria were met: FDR ≤ 0.05 and FC ≥ 2 or ≤ -2.

**Fig S1. Effects of MRME treatments on HUVECs viability.** HUVECs were treated at different MRME concentrations for 72 hours and viability was evaluated by MTT assay. Untreated sHUVECs were used as reference in the study. The data represent the mean values of three independent experiments. Error bands represent standard deviation.


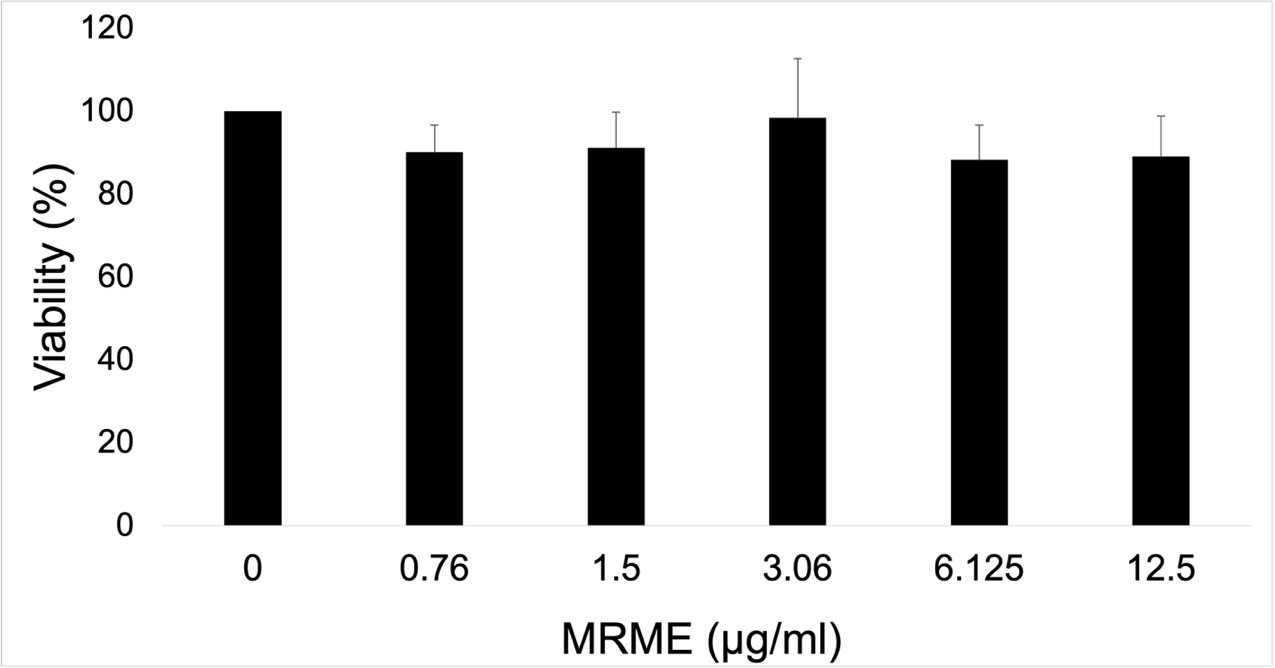


**Table S3: Transcriptomic analysis results**. List of differentially expressed genes (DEGs) obtained comparing senescent HUVECs (sHUVECs) untreated and treated with Mela Rosa Marchigiana callus extract (MRME). 11 genes were up regulated and 15 genes were downregulated in sHUVEC MRME. Genes were identified as DEGs when the following criteria were met: FDR ≤ 0.05 and FC ≥ 1.5 or ≤ -1.5.

| **GeneName** | **FC** | **logFC** | **FDR** | **Tras_ID** |
| --- | --- | --- | --- | --- |
| RASD1 | 2.43232674 | 1.28233705 | 7.4999E-08 | ENSG00000108551.4 |
| GTPBP6 | 2.22379949 | 1.15302672 | 0.00938402 | ENSGR0000178605.8 |
| HYPK | 2.03790606 | 1.02708755 | 0.02101529 | ENSG00000242028.1 |
| ZNF230 | 1.93036227 | 0.94887162 | 0.02513974 | ENSG00000159882.8 |
| PRDM1 | 1.9223511 | 0.94287185 | 0.03189461 | ENSG00000057657.10 |
| RP11-33B1.1 | 1.85957503 | 0.89497296 | 0.0085504 | ENSG00000245958.2 |
| C19orf68 | 1.85554675 | 0.89184435 | 0.02840498 | ENSG00000185453.8 |
| WDR5B | 1.62247632 | 0.69819743 | 0.04410006 | ENSG00000196981.2 |
| ZC3HAV1L | 1.56251998 | 0.64387464 | 0.02488436 | ENSG00000146858.7 |
| SEMA6D | 1.54324401 | 0.62596619 | 0.02046903 | ENSG00000137872.11 |
| LYST | 1.53293775 | 0.61629911 | 0.00226989 | ENSG00000143669.9 |
| GADD45B | 0.64753955 | -0.6269598 | 1.6667E-16 | ENSG00000099860.4 |
| DUSP1 | 0.64726647 | -0.6275683 | 8.4214E-26 | ENSG00000120129.5 |
| SOCS3 | 0.63422732 | -0.6569281 | 8.119E-11 | ENSG00000184557.3 |
| JUNB | 0.63154877 | -0.6630339 | 3.6939E-13 | ENSG00000171223.4 |
| ZFP36 | 0.59042179 | -0.7601821 | 2.1287E-09 | ENSG00000128016.4 |
| FOS | 0.57349019 | -0.8021593 | 1.1722E-17 | ENSG00000170345.5 |
| NR4A1 | 0.54984418 | -0.8629053 | 7.3596E-06 | ENSG00000123358.15 |
| HES1 | 0.51299762 | -0.962976 | 2.1149E-20 | ENSG00000114315.3 |
| ID1 | 0.44667004 | -1.1627186 | 3.8519E-47 | ENSG00000125968.7 |
| ATF3 | 0.42248007 | -1.2430448 | 4.0286E-29 | ENSG00000162772.12 |
| RABGEF1 | 0.4196816 | -1.2526329 | 0.01186952 | ENSG00000154710.11 |
| BOLA2B | 0.37540602 | -1.4134763 | 0.00534688 | ENSG00000169627.7 |
| FOSB | 0.34248538 | -1.5458857 | 7.9483E-28 | ENSG00000125740.9 |
| ID2 | 0.3332742 | -1.5852185 | 1.1603E-11 | ENSG00000115738.5 |
| KLF4 | 0.3080516 | -1.6987561 | 1.9549E-18 | ENSG00000136826.10 |

***Supplementary results: Effect of MRME on DNA methylation in sHUVEC***

To explore potential epigenetic mechanisms underlying MRME-induced gene expression changes we investigated DNA methylation in the promoter region of the 12 genes (ID2, GADD45B, JUNB, HES1, ATF3 NR4A1, KLF4, FOS, FOSB, ZFP36, DUSP1 and SOCS3) identified as differentially expressed in sHUVECs treated with MRME compared to untreated senescent cells and implicated in the TNF-α via NFKB signalling. Pyrosequencing analysis was conducted on yHUVECs, sHUVECs and sHUVECs MRME to assess the DNA methylation levels across the three conditions. The analysis revealed no significant differences in the mean methylation levels of the promoter regions of the selected genes between yHUVECs, sHUVECs and sHUVECs MRME (Figure S2). However, when the analysis was extended to single CpG sites, we found that the methylation level of the first CpG site in the selected area of SOCS3 promoter region significantly changes between the three conditions (Overall p-value = 0.009 ANOVA). In particular, the methylation level of this CpG decreases with senescence (p < 0.05), while is increased by MRME treatments of sHUVECs (p < 0.05) (Figure 5). To further investigate whether MRME influences DNA methylation levels, we extended our analysis by performing Reduced Representation Methylation Sequencing (RRMS) using PromethION24 (Oxford Nanopore Technology, UK) on DNA extracted from three biological replicates of sHUVECs and sHUVECs MRME. While pyrosequencing provides quantification of DNA methylation at specific CpG sites, it is limited by its targeted design, allowing analysis only within short, pre-amplified regions, typically 100 base pairs, of selected promoters. As a result, it cannot detect methylation changes outside the predefined loci, nor capture broader epigenetic alterations across the genome. Therefore, we employed Nanopore adaptive sampling RRMS protocol to obtain a broader, genome-wide perspective, specifically enriching for CpG-dense regions, and to detect potential epigenetic changes beyond the predefined promoter targets.

The average coverage across all the samples was approximately 20x per sample. Differential methylation analysis was firstly performed at CpGs locus level. No significant differences in DNA methylation levels were observed after adjustment for multiple comparisons comparing sHUVECs and sHUVECs MRME in any CpG site (data not shown) . Similarly, no significant differences were detected when the analysis was extended to promoter regions (Figure S3).

**Fig. S2 Analysis of the methylation status in the promoter regions of the 12 selected genes.** Average methylation level measured by Pyrosequencing analysis in the selected area of the ID2 promoter (A), GADD45B promoter (B), JUNB promoter (C), HES1 promoter (D), ATF3 promoter (E), NR4A1 promoter (F), KLF4 promoter (G), FOS promoter (H), FOSB promoter (I), ZFP36 promoter (L), DUSP1 promoter (M), SOCS3 promoter (N) in the three groups: Y (young HUVECs), S (senescent HUVECs), M (Senescent HUVECs treated with the Mela Rosa Marchigiana callus extract). There was no significant difference in the mean methylation levels of the promoter regions of the selected genes comparing yHUVECs, sHUVECs and sHUVECs MRME. The boxes represent the interquartile range (25th–75th percentile), the line inside the box indicates the median, and the whiskers extend to the smallest and largest values within 1.5 times the interquartile range.


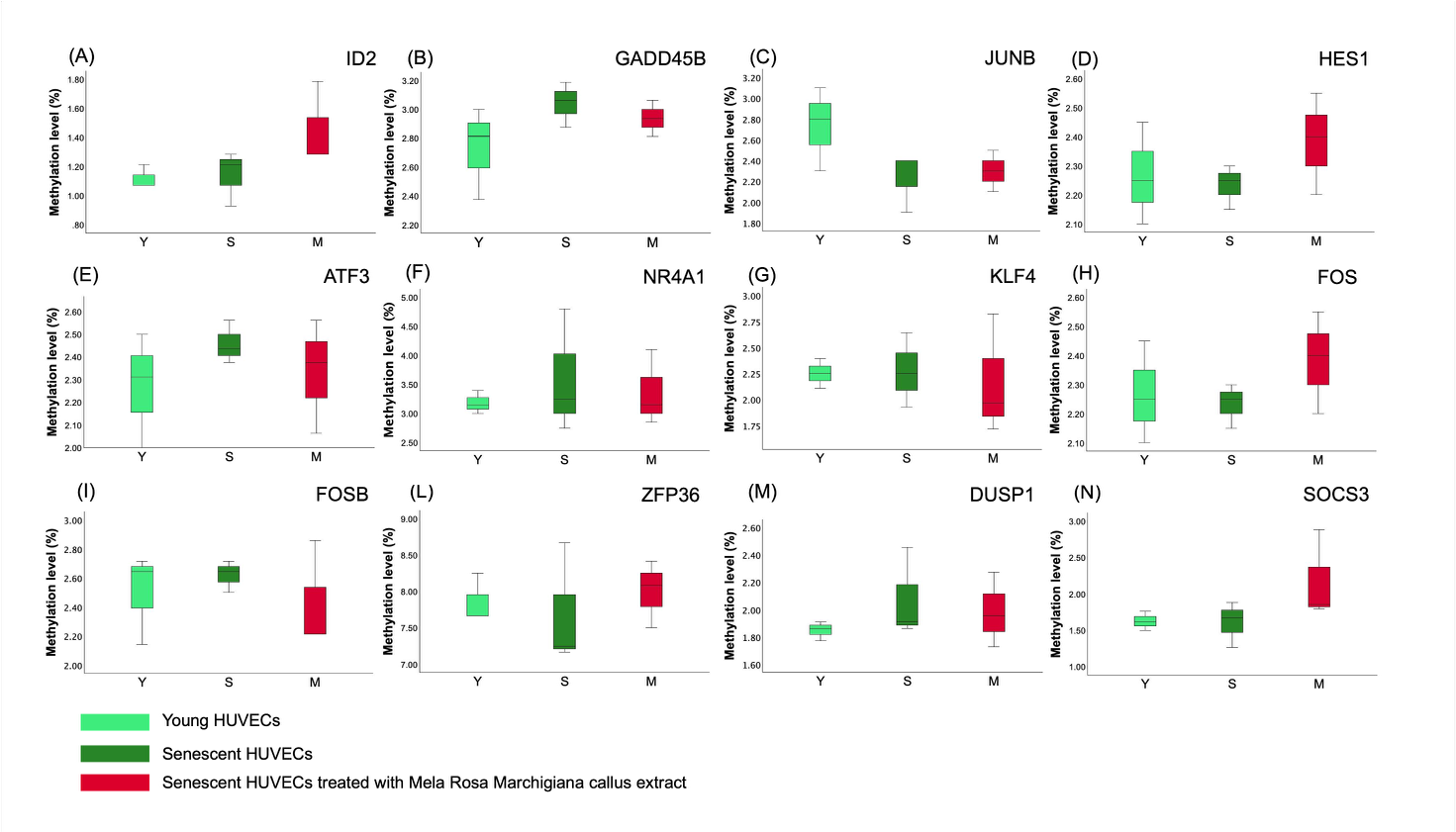


**Fig. S3: Analysis of genome-wide methylation by Oxford Nanopore sequencing (RRMS).** MD plot showing the log-fold-change of the methylation level in gene promoters, representing the difference between senescent HUVECs treated with MRME and untreated senescent HUVECs, plotted against the average CpG abundance within each gene promoter. No significant differences in DNA methylation levels were observed between groups after correction for multiple testing (Bonferroni correction). Promoters not significantly differentially methylated (FDR > 0.05) are shown in black.


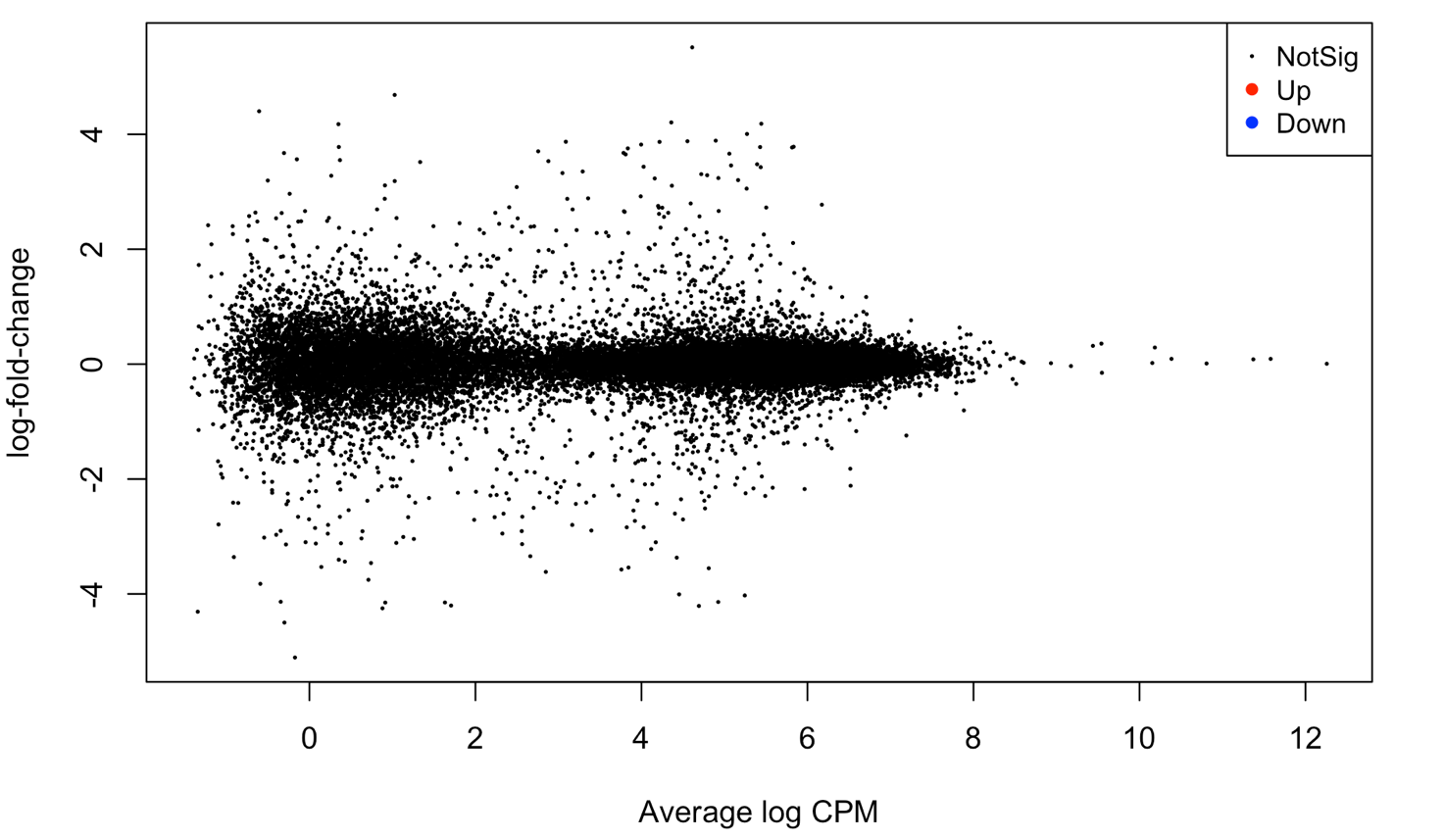

Supplement: Supplementary file 1 — Supporting Information File 1: mnfr70336‐sup‐0001‐SuppMat.docx. [file MNFR-69-e70336-s002.docx]
